# Breast health information, motivation, behavioral skills, and breast self-examination behavior among female medical college students: a cross-sectional study

**DOI:** 10.3389/fpubh.2026.1965648

**Published:** 2026-10-13

**Authors:** Die Wu, Li Meng

**Affiliations:** Zhangzhou Health Vocational College, Zhangzhou, Fujian, China

**Keywords:** breast cancer prevention, breast self-examination, cross-sectional study, health education, information-motivation-behavioral skills model, self-efficacy

## Abstract

**Objective:**

Based on the Information-Motivation-Behavioral Skills Model (IMB), this study examined differences in breast cancer (BC) prevention knowledge, perceived barriers, self-efficacy, and breast self-examination (BSE) practices between female nursing and pharmacy students. We hypothesized that nursing students would demonstrate more favorable IMB profiles and that the association between academic major and BSE behavior would be statistically explained by these constructs.

**Methods:**

A cross-sectional survey was conducted among 997 female nursing and pharmacy students from a medical college (May-June 2026). Data were collected using the Champion Health Belief Model Scale (CHBMS) and a self-developed BC knowledge questionnaire. Multivariate logistic regression identified factors associated with BSE behavior, adjusting for grade, residence, and family history. Analyses were performed using SPSS 20.0 (α = 0.05).

**Results:**

Compared with pharmacy students, nursing students exhibited significantly higher knowledge scores (7.21 ± 2.51 vs. 6.05 ± 2.89, *t* = 6.21, *p* < 0.001, Cohen's d = 0.43), self-efficacy scores (3.35 ± 0.72 vs. 3.18 ± 0.79, *t* = 3.54, *p* < 0.001, Cohen's d = 0.22), and BSE rates (60.6% vs. 32.0%, χ^2^ = 81.07, *p* < 0.001), with small to moderate effect sizes. Nursing students also reported lower perceived barrier scores (2.65 ± 0.78 vs. 2.95 ± 0.85, *t* = −6.04, *p* < 0.001, Cohen's d = −0.37). Multivariate logistic regression identified prior breast health education (OR = 4.642, 95% CI: 3.231–6.670, *p* < 0.001) and higher self-efficacy (OR = 1.817, 95% CI: 1.453–2.272, *p* < 0.001) as independent predictors of BSE, while higher perceived barriers were associated with lower odds of BSE (OR = 0.286, 95% CI: 0.226–0.360, *p* < 0.001). After adjusting for these IMB constructs, the OR for nursing major decreased from 1.912 to 1.648, suggesting that these factors partially explain the inter-major difference in BSE behavior.

**Conclusion:**

In this cross-sectional study, nursing students demonstrated higher BC prevention literacy than pharmacy students. Higher self-efficacy and lower perceived barriers, but not knowledge, were independently associated with BSE. These findings suggest that breast health education in medical colleges should prioritize skills training and barrier reduction over knowledge transfer alone.

## 1 Introduction

The latest estimates from the Global Cancer Observatory (GLOBOCAN 2022, version 1.1, released in February 2024) indicate that approximately 2,296,840 women worldwide were newly diagnosed with BC in 2022, accounting for 23.8% of all female cancer cases. During the same period, 666,103 women died from the disease, accounting for 15.5% of female cancer-related deaths (1). In China, 357,200 new BC cases were reported in 2022, making it the sixth most common malignancy among all cancers and the second most common malignancy among women, after lung cancer. The female-specific age-standardized incidence rate (ASIR) was 35.3/100,000 based on the Chinese population standard and 33.0/100,000 based on the world standard population (2). In the same year, approximately 75,000 women died from BC, accounting for 2.9% of all cancer-related deaths and 7.9% of female cancer-related deaths in China (2). Moreover, the incidence of BC among young Chinese women aged 15–39 years has shown a continuous increasing trend from 1990 to 2021. According to a study by Sheng Y et al. (2025), the ASIR of BC among young Chinese women increased from 6.50 per 100,000 in 1990 to 12.08 per 100,000 in 2021, corresponding to an average annual percent change (AAPC) of 2.0% (*p* < 0.001). Notably, the increasing trend accelerated after 2015, with an annual percent change of 3.5% (3). This shift toward a younger age at onset highlights female college students as a population requiring greater attention regarding BC prevention and risk awareness. In China, the 5-year survival rate of patients with BC has increased to 83%, approaching the rates reported in several developed countries (4). BSE is a simple, low-cost, and accessible approach for detecting breast abnormalities. However, international screening recommendations regarding routine BSE remain inconsistent. Although major guidelines acknowledge that BSE may increase the detection of breast lumps, evidence from large-scale trials has not demonstrated a corresponding reduction in BC mortality (5). Therefore, it is important to distinguish between the role of BSE in identifying breast abnormalities and its potential impact on reducing BC mortality. While BSE can promote breast awareness and serve as a complementary approach, it should not be considered a standalone strategy for secondary prevention of BC. Despite its recognized benefits, BSE practice remains limited among female college students in China. A cross-sectional survey was conducted in 2021 among 2,400 female college students from 15 comprehensive universities in Anhui Province (the manuscript was received in May 2020). In this study, “awareness” was defined as a score ≥60% of the total score on the BSE knowledge section, resulting in an overall awareness rate of 73.62%. “Regularly performing BSE” was defined as a score ≥60% of the total score on the behavior section, with an overall implementation rate of 27.91%. According to the screening guidelines referenced in the study, which recommended monthly self-examination for women aged 20 years and above, “regularly performing” is generally interpreted as conducting BSE at least once per month. Although 73.62% of participants demonstrated awareness of relevant breast health knowledge, only 27.91%−29.82% regularly practiced BSE, indicating a substantial gap between knowledge acquisition and actual health behavior (6). The IMB is a widely applied framework for understanding and predicting health-related behaviors. Introduced in 1992, the model proposes that the adoption of health behaviors is determined by three core components: information, motivation, and behavioral skills (7). According to the IMB model, information and motivation are generally considered to influence the initiation and maintenance of health behaviors through behavioral skills. Specifically, the model proposes that behavioral skills mediate the relationship between information/motivation and behavior, such that individuals with greater knowledge and stronger motivation are more likely to develop the skills required to perform the behavior. However, the model also recognizes that information and motivation may have direct effects on behavior under certain circumstances, such as when behavioral skills are relatively simple or when the behavior is newly adopted. In the context of BSE, based on previous studies applying the IMB model to this area, we hypothesized that BSE-related information and motivation would influence BSE practices through both direct and indirect pathways, with BSE-related behavioral skills serving as the primary proximal determinant of BSE behavior. In Fujian Province, the incidence rate of BC exceeds the national average. According to Wen et al. (based on data from 2021to 2020), the ASIR in Fujian increased from 19.34/100,000 to 38.73/100,000, with an AAPC of 7.4%, which was substantially higher than the national average increase of 3%−4%. The 2022 Fujian Cancer Registry Annual Report further indicated that the incidence rate in Fujian was 41.37/100,000, compared with the national rate of 39/100,000. In addition, the proportion of younger patients (aged < 40 years) has continued to increase, accounting for approximately 15% of newly diagnosed BC cases in Fujian, with patients aged < 35 comprising 6% to 7%. The increasing trend highlights the urgent need to strengthen early detection and timely treatment strategies, particularly among younger women (8). Accordingly, this study adopted the IMB model as its theoretical framework to evaluate BC prevention knowledge and its association with BSE behavior among female students at Zhangzhou Health Vocational College. Within this framework, the three core constructs were operationalized as: (1) information was assessed using a self-developed knowledge questionnaire that evaluated understanding of BC risk factors, early warning signs, and BSE procedures; (2) motivation was operationalized as perceived barriers to performing BSE, including insufficient knowledge of correct technique, embarrassment, and forgetfulness, which were assessed using the perceived barriers subscale of Champion Health Belief Model Scale (CHBMS); and (3) behavioral skills were operationalized as self-efficacy in performing BSE, which was assessed using the self-efficacy subscale of the CHBMS. Perceived barriers and self-efficacy were selected as the primary indicators of motivation and behavioral skills, respectively, because they represent proximal and modifiable determinants of health behavior within the IMB framework and have been consistently identified as important predictors of BSE in previous studies. The outcome variable, BSE behavior, was defined as regular practice performed at least once per month, consistent with screening guideline recommendations. This study employed a cross-sectional survey design, with regular BSE practice among female college students defined as the primary outcome. The findings suggest that students with lower knowledge levels, higher perceived barriers, or lower self-efficacy, particularly those characteristics more commonly observed among non-nursing students, may benefit most from targeted interventions. Given the cross-sectional nature of this study, these associations require further validation through experimental designs. The findings are expected to provide empirical support for the development of targeted and stratified breast health education interventions in colleges and universities.

## 2 Materials and methods

### 2.1 Study population

This cross-sectional study investigated BC prevention knowledge and BSE behavior among female students at Zhangzhou Health Vocational College. This study included full-time female students enrolled in nursing and pharmacy programs at Zhangzhou Health Vocational College between May and June 2026. A one-stage cluster sampling method was employed, with classes used as the sampling units. The sampling frame comprised 24 classes (12 nursing and 12 pharmacy), from which classes were randomly selected using simple random sampling. All female students in the selected classes were invited to participate. Although the exact number of selected classes and the total number of eligible students within those clusters were not formally recorded, the final analytic sample included 538 nursing and 459 pharmacy students. Since the survey was conducted anonymously and class identifiers were not retained in the final dataset, the specific class-level response structure could not be reconstructed for analysis. Therefore, individual students were treated as the unit of analysis, and design effects or cluster-adjusted variance estimation were not applied. Potential intra-class correlation due to shared educational experiences among students within the same class may have resulted in slightly underestimated standard errors. This limitation should be considered when interpreting the precision of the reported estimates. Due to voluntary participation and clinical internships, particularly among 3-year students, some students declined participation or were unavailable, resulting in unit non-response. Female students from the selected classes completed the survey using the Wenjuanxing online questionnaire platform. All participants completed the questionnaires independently after class without on-site supervision. The inclusion criteria were as follows: 1) full-time female students currently enrolled in the nursing or pharmacy programs at Zhangzhou Health Vocational College, as verified through official college registration records; and 2) voluntary participation with informed consent provided through the completion and submission of the questionnaire. The exclusion criteria were as follows: (1) students majoring in disciplines other than nursing or pharmacy; (2) students with a self-reported previous diagnosis of BC or any other breast-related disease (e.g., benign breast lesions and mastitis); (3) questionnaires with incomplete data (e.g., missing responses for key variables) or obvious response inconsistencies (e.g., contradictory answers or patterned responses); and (4) students who were unable or unwilling to provide informed consent. The questionnaire was administered through the Wenjuanxing platform. Before completing the survey, participants were informed of the study objectives and assured that their responses would remain anonymous. Completion and submission of the questionnaire were considered as providing informed consent, and students who declined participation were permitted to withdraw without completing the survey. A total of 1,014 questionnaires were collected, of which 997 were considered valid, including 538 responses from nursing students and 459 responses from pharmacy students. The 17 excluded questionnaires were from students in non-target disciplines, including 6 from other medicine-related fields and 11 from non-medical majors. The overall valid response rate was 98.32%. Ethical approval for this study was obtained from the Ethics Committee of Zhangzhou Health Vocational College (ZWY2026014). The electronic informed consent procedure, in which participants provided consent by clicking “Start” and completing the questionnaire after reviewing a cover letter describing the study purpose, anonymity, and voluntary participation, was reviewed and approved as part of the initial ethical review process. To prevent duplicate submissions, the online survey platform was configured to allow only one response per IP address and device. Because the survey was anonymous, individual responses could not be identified or removed after submission. This limitation was stated in the cover letter.

### 2.2 Research instruments

#### 2.2.1 Breast health belief scale

Based on the IMB theoretical framework, this study adopted two dimensions from CHBMS (9). Within the IMB framework, motivation encompasses personal attitudes, beliefs, and perceived social support that influence an individual's willingness to adopt a health behavior, with perceived barriers representing an important negative component. The IMB model conceptualizes personal motivation as including beliefs regarding intervention outcomes and attitudes toward specific health behaviors, whereas social motivation involves perceived social support and social norms (10). Therefore, the CHBMS perceived barriers subscale, including items related to insufficient knowledge of correct techniques and embarrassment, was mapped to the motivation construct. Behavioral skills refer to both the objective abilities and perceived self-efficacy required to perform a health behavior (11). The IMB model identifies self-efficacy as a core component of behavioral skills, and empirical studies have consistently demonstrated that self-efficacy is a significant direct determinant of health behavior and can mediate the effects of information and motivation on behavioral outcomes (12). Therefore, the CHBMS self-efficacy subscale was considered a conceptually appropriate indicator of the behavioral skills construct. Although the CHBMS was originally developed based on the Health Belief Model (HBM), the cross-sectional integration of HBM constructs into the IMB framework has been supported by previous studies. For example, the IMB model has been applied to health behaviors such as waste separation, where environmental knowledge was integrated to explain behavioral outcomes (13), and to HPV vaccination studies, where health beliefs and perceived barriers were incorporated as components of motivation (14). The remaining CHBMS dimensions, including perceived susceptibility, perceived severity, and perceived benefits, were not included in this study because they represent more distal cognitive antecedents of behavior rather than the proximal motivational and skill-related determinants emphasized by the IMB framework (14). These two dimensions were selected because they represent the most proximal and modifiable determinants of health behavior change within the IMB framework. In contrast, the remaining CHBMS dimensions, including perceived susceptibility, severity, and benefits, reflect more distal cognitive antecedents. The perceived barriers dimension included three items: “I do not know the correct method for performing BSE,” “I feel embarrassed about performing BSE,” and “I often forget to perform BSE.” The self-efficacy dimension comprised two items: “I am confident that I can complete BSE according to the correct procedure” and “I can continue performing monthly BSE even without reminders.” Each item was assessed using a five-point Likert scale ranging from 1 (strongly disagree) to 5 (strongly agree). Each item included three response options: “True,” “False,” and “Don't know.” For positively worded items, correct responses (“True”) were assigned a score of 1, whereas incorrect responses (“False”) or “Don't know” responses were assigned a score of 0. Two reverse-scored items were included to assess critical health literacy: K6 (“BSE can replace professional clinical breast examination”) and K10 (“Men do not get BC, so they do not need to pay attention to breast health”). For these items, “False” responses were assigned a score of 1, whereas “True” or “Don't know” responses were assigned a score of 0. For the perceived barriers subscale (3 items; score range: 3–15), higher scores indicated greater perceived barriers to performing BSE. For the self-efficacy subscale (2 items; score range: 2–10), higher scores indicated greater confidence in performing BSE. Both subscales were analyzed using their original scoring directions, and no score reversal was performed. Higher scores reflected greater perceived barriers or higher self-efficacy. In the pilot investigation (*n* = 43), the Cronbach's alpha coefficients for the perceived barriers and self-efficacy dimensions were 0.593 and 0.721, respectively. The relatively low internal consistency of the perceived barriers subscale was addressed by refining the Chinese wording of the three items to improve clarity before the main survey. In the formal survey (*n* = 997), the Cronbach's alpha coefficients increased to 0.79 and 0.81, respectively, indicating satisfactory internal consistency. The Chinese version of the CHBMS used in this study was adapted from the version validated by Wan et al. (9), which underwent formal translation, back-translation, and psychometric evaluation among Chinese stroke patients. Based on the IMB model, the perceived barriers (3 items) and self-efficacy (2 items) subscales were selected for assessment. In the pilot test (*n* = 43), Cronbach's α for these subscales was 0.593 and 0.721, respectively. The relatively low reliability of the perceived barriers subscale was addressed by further refining the wording of the three items based on feedback from the pilot study to improve their suitability for the target population of young female students. In the main survey (*n* = 997), the Cronbach's α values increased to 0.79 and 0.81, demonstrating satisfactory internal consistency. The self-developed knowledge questionnaire was developed based on established guidelines and relevant literature. The questionnaire items were subsequently refined through discussions among the research team, which included two breast surgeons (associate chief physicians), one associate professor of public health and epidemiology, and two clinical medicine instructors (associate professors), as well as feedback from the pilot study (*n* = 43), thereby ensuring appropriate content validity.

#### 2.2.2 Breast cancer prevention and control knowledge scale

The knowledge questionnaire was developed based on the *Guidelines for Breast Cancer Diagnosis and Treatment* by the China Anti-Cancer Association (2026 Edition) (5) and relevant literature. No items were adapted from previously validated instruments because no existing validated Chinese BC knowledge questionnaire was identified that adequately addressed the specific knowledge domains relevant to young female college students. Content validity was established through expert review, and internal consistency was evaluated using the KR-20 coefficient, which was 0.913 in the main survey. However, additional psychometric assessments, including construct validity (e.g., exploratory or confirmatory factor analysis), criterion-related validity evaluations, and item-level analyses (e.g., item difficulty and discrimination indices), were not performed. This limitation should be considered when interpreting the knowledge-related findings. Further validation of the questionnaire among larger and more diverse student populations is warranted. The content validity of the initial item pool was assessed by an expert panel comprising two breast surgeons (associate chief physicians), one associate professor of public health and epidemiology, and two clinical medicine instructors (associate professors). The items were subsequently refined through research team discussions and feedback obtained from the pilot study (*n* = 43). The scale consisted of 10 items covering key aspects of BC prevention and control, including epidemiology, risk factors, early symptoms, screening approaches, and BSE-related knowledge. For each item, participants selected one of three responses: “True,” “False,” and “Don't know.” Each correct response was assigned 1 point, whereas incorrect responses and “Don't know” responses were assigned 0 points. The total knowledge score ranged from 0 to 10, with higher scores indicating greater BC prevention and control knowledge. The reliability of the questionnaire was evaluated using the Kuder–Richardson Formula 20 (KR-20), which assesses the internal consistency of dichotomously scored items. The KR-20 coefficient was 0.887 in the pilot study and 0.89 in the formal survey, indicating good internal consistency. In addition to content validity established through expert review, no further construct validity or criterion validity analyses were conducted.

#### 2.2.3 General characteristics and BSE behavior

The collected general characteristics included school, grade level, academic major, place of origin, family residence, family history of BC, and previous experience with breast health knowledge acquisition. BSE behavior was assessed using a single question: “Have you ever performed BSE?” Responses were dichotomized, with “Yes” assigned a value of 1 and “No” assigned a value of 0. Although this dichotomous measure is commonly used in BSE research, it does not assess the regularity, frequency, or accuracy of BSE practice. This limitation should be considered when interpreting the findings. Future studies are recommended to collect additional information regarding monthly BSE adherence and self-examination technique to provide a more comprehensive evaluation of BSE behavior.

### 2.3 Statistical analysis

All statistical analyses were performed using SPSS 20.0. The normality of continuous variables was assessed using the Shapiro-Wilk test before conducting parametric analyses. Student's *t*-test was applied for variables with equal variances, whereas Welch's *t*-test was used for variables that did not meet the assumption of equal variances. Continuous variables between the two groups were compared using the independent-samples *t*-test, and categorical variables were analyzed using Pearson's chi-square test. Effect sizes for between-group comparisons were calculated using Cohen's d, with values of 0.20, 0.50, and 0.80 representing small, medium, and large effects, respectively. Continuous variables were reported as mean ± standard deviation, and categorical variables were presented as frequencies and percentages. BSE behavior was defined as the dependent variable and coded as Yes = 1 and No = 0. The independent variables included academic major (nursing = 1, pharmacy = 0), grade level (senior = 1, junior = 0), previous experience with breast health knowledge learning (Yes = 1, No = 0), family history (Yes = 1, No = 0), family residence (with urban residence as the reference category and rural and township residence included as two dummy variables), total knowledge score, self-efficacy, and perceived barriers. Covariates were selected based on the IMB theoretical framework and previous empirical evidence. Knowledge (information), perceived barriers (motivation), and self-efficacy (behavioral skills) were included as the primary predictors of interest. Grade level, place of residence, and family history of BC were included as clinically relevant potential confounders regardless of their statistical significance in univariate analyses. Binary logistic regression analysis was conducted using the Enter method to estimate ORs and corresponding 95% confidence intervals (95% CIs). All 997 valid cases were included in the model, with no missing data identified. Therefore, a complete-case approach was applied. Multicollinearity among independent variables was evaluated using the variance inflation factor (VIF) and tolerance values. All VIF values were close to 1.0 (1.044–1.054), and all tolerance values were greater than 0.94, indicating no significant multicollinearity. Model fit was assessed using the Hosmer-Lemeshow goodness-of-fit test, and the overall explanatory power was evaluated with Nagelkerke R^2^. Interaction terms among IMB constructs and formal mediation analyses were not included in the final regression model because this cross-sectional study primarily focused on evaluating main effects and could not establish causal mediation pathways. Model fit was assessed using the Hosmer–Lemeshow test, goodness-of-fit test for calibration, Nagelkerke R^2^ for explanatory power, and the area under the ROC curve (AUC/C-statistic) for discrimination. Statistical significance was determined using a two-sided *p* < 0.05. Considering the exploratory nature of the univariate analyses, no formal adjustment for multiple comparisons (e.g., Bonferroni correction) was performed. The primary conclusions were based on the multivariate logistic regression model, which included simultaneous adjustment for all covariates.

## 3 Results

### 3.1 Baseline demographic characteristics

The baseline characteristics of the 997 participants are summarized in Table 1. The study population was predominantly freshmen, who accounted for nearly three-quarters of the sample. In terms of residence, participants from county/town areas constituted the largest group (43%), followed by those from rural areas (35.6%) and urban areas (21.3%). Notably, only 10% of participants had a confirmed family history of breast cancer, while the majority (71.8%) had previously received breast health education.

**Table 1:** Baseline demographic characteristics of the study sample (*N* = 997).

Characteristic		*n*	%
Grade	Freshman	742	73.2
	Sophomore	234	23.1
	Junior	37	3.7
Place of residence	Urban	216	21.3
	County/town	436	43
	Rural	361	35.6
Family history of breast cancer	Yes	101	10
	No/uncertain	896	90
Prior breast health education	Yes	727	71.8
	No	286	28.2

### 3.2 Comparison of baseline characteristics between the two groups

This study included 538 nursing students and 459 pharmacy students. As shown in Table 2, nursing students had significantly higher scores for BC prevention and control knowledge [7.21 ± 2.51 vs. 6.05 ± 2.89, mean difference = 1.16, 95% CI: (0.72, 1.41), Welch's *t* = 6.098, df = 873.23, *p* < 0.001] and self-efficacy [3.35 ± 0.72 vs. 3.18 ± 0.79, mean difference = 0.17, 95% CI: (0.08, 0.27), *t* = 3.54, df = 995, *p* < 0.001] than pharmacy students. In contrast, nursing students reported significantly lower perceived barrier scores than pharmacy students [2.65 ± 0.78 vs. 2.95 ± 0.85, mean difference = −0.30, 95% CI: (−0.40, −0.21), *t* = −6.04, df = 995, *p* < 0.001]. In contrast, nursing students reported significantly lower perceived barrier scores than pharmacy students (2.65 ± 0.78 vs. 2.95 ± 0.85, df = 995, *t* = −6.04, *p* < 0.001).

**Table 2:** Comparison of knowledge, self-efficacy, and perceived barriers between nursing and pharmacy students.

Variable	Nursing (*n* = 538)	Pharmacy (*n* = 459)	*t*	*P* value	Cohen's d
Total knowledge score	7.21 ± 2.51	6.05 ± 2.89	6.21	< 0.001	0.43
Self-efficacy	3.35 ± 0.72	3.18 ± 0.79	3.54	< 0.001	0.22
Perceived barriers	2.65 ± 0.78	2.95 ± 0.85	−6.04	< 0.001	−0.37

### 3.3 univariate Analysis of BSE behavior

As presented in Table 3, Pearson's chi-square test showed that nursing students had a significantly higher proportion of BSE practice than pharmacy students (60.6% vs. 32.0%, χ^2^ = 81.07, *p* < 0.001, Cramér's V = 0.285). This finding suggests a moderate association between academic major and BSE behavior. However, this univariate association should be interpreted cautiously because it does not account for potential confounding factors, such as prior breast health education or grade level. The independent associations were further evaluated using the multivariate regression analysis.

**Table 3:** Comparison of BSE rates between the two groups.

Major	Performed BSE, *n* (%)	Did not perform BSE, *n* (%)	Total
Nursing	326 (60.6)	212 (39.4)	538
Pharmacy	147 (32.0)	312 (68.0)	459
Total	473 (47.4)	524 (52.6)	997

χ^2^ = 81.07, *P* < 0.001. Perceived barriers scores were analyzed using their original direction; therefore, OR < 1 indicates that higher perceived barriers were associated with lower odds of BSE practice.

### 3.4 Multivariate binary logistic regression analysis of BSE behavior

BSE behavior was defined as the dependent variable, and binary logistic regression analysis was performed using the Enter method. A total of 997 cases were included in the final logistic regression model, with no cases excluded because of missing data. In the basic model without adjustment for mediating variables, the results (Table 4) showed that nursing major (OR = 1.912, 95% CI: 1.416–2.583, *p* < 0.001), senior grade level (OR = 1.432, 95% CI: 1.034–1.982, *p* = 0.031), and previous learning experience related to breast knowledge (OR = 4.642, 95% CI: 3.231–6.670, *p* < 0.001) were significant independent factors associated with a higher likelihood of BSE behavior. In contrast, family history and family residence were not significantly associated with BSE behavior (*p* > 0.05). The logistic regression model demonstrated satisfactory overall performance. The model was statistically significant (χ^2^ = 345.329, df = 6, *p* < 0.001), and calibration was adequate, as indicated by a non-significant Hosmer-Lemeshow test (χ^2^ = 8.243, df = 8, *p* = 0.410).

**Table 4:** Binary Logistic regression analysis of factors associated with BSE behavior (basic model).

Variable	B	SE	OR (95% CI)	*p* value
Nursing major (vs. pharmacy)	0.648	0.153	1.912 (1.416–2.583)	< 0.001
Senior grade (vs. junior grade)	0.359	0.467	1.432 (1.034–1.982)	0.031
Previous learning experience related to breast knowledge (yes vs. no)	1.535	0.185	4.642 (3.231–6.670)	< 0.001
Family history (yes vs. no)	0.281	0.247	1.325 (0.819–2.144)	0.252
Rural residence (vs. urban)	0.172	0.190	1.187 (0.818–1.723)	0.367
Township residence (vs. urban)	−0.149	0.535	0.862 (0.698–1.064)	0.169

B unstandardized regression coefficient; SE, standard error; OR, odds ratio; CI, confidence interval; Ref., reference category. The basic model included academic major, grade level, prior breast health education, family history of BC, and place of residence. Model performance: Hosmer-Lemeshow χ^2^ = 9.452, df = 8, *p* = 0.306; Nagelkerke R^2^ = 0.218; AUC = 0.725. Perceived barriers scores were analyzed using their original direction; therefore, OR < 1 indicates that higher perceived barriers were associated with lower odds of BSE practice.

After further adjustment for total knowledge score, self-efficacy, and perceived barriers (extended model, Table 5), the OR for nursing major decreased from 1.912 to 1.648 (95% CI: 1.162–2.336, *p* = 0.005). This attenuation suggests that these IMB constructs may statistically explain part of the difference in BSE behavior between nursing and pharmacy students. However, this finding should be interpreted cautiously, as a reduction in OR after covariate adjustment alone does not provide evidence of mediation. Formal mediation analysis using appropriate methods, such as bootstrapped indirect effects or structural equation modeling, was not performed in this cross-sectional study. Future longitudinal or experimental studies using these approaches are needed to rigorously evaluate potential mediating pathways. However, because formal mediation analysis was not conducted in this cross-sectional study, these findings should be interpreted as observational associations rather than evidence of causal mediation. Previous learning experience related to breast knowledge (OR = 2.766, 95% CI: 1.841–4.158, *p* < 0.001), total knowledge score (OR = 1.200, 95% CI: 1.127–1.279, *p* < 0.001), self-efficacy (OR = 1.817, 95% CI: 1.453–2.272, *p* < 0.001), and perceived barriers (OR = 0.286, 95% CI: 0.226–0.360, *p* < 0.001) remained significant independent predictors of BSE behavior. In addition, family history (OR = 0.286, *p* < 0.001) was significantly associated with a lower likelihood of performing BSE, whereas grade level and family residence were not associated with BSE behavior (*p* > 0.05).

**Table 5:** Binary logistic regression analysis of factors associated with BSE behavior (extended model).

Variable	B	SE	Wald	OR (95% CI)	*p* value
Nursing major (vs. pharmacy)	0.499	0.153	17.869	1.648 (1.162–2.336)	0.005
Previous learning experience related to breast knowledge (yes vs. no)	1.018	0.185	68.010	2.766 (1.841–4.158)	< 0.001
Total knowledge score	0.183	0.032	0.433	1.200 (1.127–1.279)	< 0.001
Self-efficacy	0.597	0.113	31.977	1.817 (1.453–2.272)	< 0.001
Perceived barriers	−1.253	0.117	135.249	0.286 (0.226–0.360)	< 0.001
Family history (yes vs. no)	−0.281	0.246	1.311	0.286 (0.226–0.360)	< 0.001
Grade (senior vs. junior)	0.228	0.166	11.660	1.256 (0.865–1.824)	0.231
Rural residence (vs. urban)	−0.017	0.190	–	0.983 (0.644–1.500)	0.936
Township residence (vs. urban)	−0.835	0.336	–	0.434 (0.147–1.283)	0.131

The identical OR value of 0.286 for family history and perceived barriers occurred coincidentally. In the extended model, family history of BC was not significantly associated with BSE behavior (OR = 0.755, 95% CI: 0.466–1.222, *p* = 0.252). Similarly, univariate analysis showed no significant association between family history and BSE behavior (χ^2^ = 1.953, *p* = 0.162), with the association direction indicating a weak positive trend (OR = 1.367, 95% CI: 0.881–2.121). The reduction in OR from 1.367 to 0.755 after adjustment for IMB constructs suggests that the initial weak positive association may have been influenced by confounding factors, including knowledge, self-efficacy, and perceived barriers. The relatively low prevalence of a positive family history in this sample (10.0%, *n* = 101) may have limited statistical power to detect a true association. These variables were independently entered into the regression model and showed distinct associations with BSE behavior. The effect estimates and confidence intervals reflect the results obtained from the analyzed data. Among the significant predictors, perceived barriers showed the strongest independent contribution (Wald = 135.249), followed by prior breast health education (Wald = 68.010), self-efficacy (Wald = 31.977), nursing major (Wald = 17.869), and grade level (Wald = 11.660). Total knowledge score (Wald = 0.433), place of residence, and family history did not contribute significantly to the model. The logistic regression model demonstrated satisfactory overall performance. The model was statistically significant (χ^2^ = 345.329, df = 6, *p* < 0.001). Calibration was adequate, as indicated by a non-significant Hosmer-Lemeshow test (χ^2^ = 8.243, df = 8,*p*= 0.410). The extended model demonstrated good discriminatory ability (AUC = 0.823) and adequate calibration (Hosmer-Lemeshow χ^2^ = 8.243, df = 8, *P* = 0.410), explaining 39.1% of the variance in BSE behavior (Nagelkerke R^2^ = 0.391). As supplementary descriptive measures, the overall classification accuracy was 74.8%, with a sensitivity of 71.7%, specificity of 77.7%, positive predictive value (PPV) of 74.3%, and negative predictive value (NPV) of 75.2%. Classification accuracy alone should be interpreted cautiously because it may be influenced by the relatively low prevalence of regular BSE in this sample (9.96%). Among the significant predictors in the extended model, perceived barriers showed the largest independent contribution (Wald = 135.249), followed by prior breast health education (Wald = 68.010), self-efficacy (Wald = 31.977), nursing major (Wald = 17.869), and grade level (Wald = 11.660). Total knowledge score (Wald = 0.433, *p* = 0.510), place of residence, and family history did not contribute significantly to the model.

## 4 Discussion

Early detection of BC is associated with greater treatment options and improved prognosis. BSE may promote breast awareness and facilitate the timely identification of breast abnormalities. Previous studies have shown that breast lumps are among the most common initial manifestations of BC (15), and many women first recognize breast changes through self-palpation. However, BSE should be distinguished from formal screening strategies, such as mammography, as its primary role is to promote breast awareness rather than serve as a population-based screening method. Current evidence from large-scale randomized trials has not demonstrated that routine BSE instruction reduces BC mortality. The landmark Shanghai trial (1992–2001), which included more than 266,000 female textile workers, found no significant difference in BC mortality between women who received intensive BSE training and those who did not, although the training group identified more benign lumps and underwent more biopsies (16). Similarly, the Russian WHO-supported trial did not demonstrate a mortality benefit associated with routine BSE (16). Consequently, major international guidelines have shifted from recommending systematic BSE training for all women toward promoting breast self-awareness, which emphasizes familiarity with one's own breasts and the ability to recognize changes. For instance, the American Cancer Society (ACS) and the U.S. Preventive Services Task Force (USPSTF) no longer recommend routine BSE instruction, whereas the National Comprehensive Cancer Network (NCCN) and the Chinese Breast Cancer Screening Guidelines (2022) continue to recognize the value of BSE in promoting awareness, particularly among populations with limited access to mammography (17). Therefore, evaluating BSE as a behavioral outcome rather than a mortality-reducing screening intervention is scientifically appropriate, as BSE represents a feasible and low-cost indicator of health awareness and preventive behavior among young women. Therefore, this study considered BSE as a component of breast health awareness rather than a standalone screening modality with established mortality benefits. In the univariate analysis of BSE behavior, nursing students demonstrated a significantly higher BSE rate than pharmacy students (60.6% vs. 32.0%, χ^2^ = 81.07, *p* < 0.001). This difference may be partly attributable to variations in the educational content and training approaches incorporated into the curricula of the two academic programs. For instance, nursing students receive structured education on breast diseases during their 2nd year through the Surgical Nursing course, which includes theoretical instruction on breast diseases and hands-on practice using human body models in the clinical training center. In contrast, although pharmacy students are enrolled in a healthcare-related discipline, they only complete the Introduction to Clinical Medicine course, in which surgical topics receive limited instructional time and breast diseases are not specifically addressed. Furthermore, pharmacy students do not receive practical training related to surgical disease procedures (18). However, this curriculum-based interpretation remains a hypothesis rather than an established mechanism because detailed information on curricular exposure, including specific course hours, clinical training intensity, and direct observation of breast health education sessions, was not systematically collected in this study. Future studies should incorporate more detailed curricular data to directly assess whether and to what extent curriculum differences contribute to variations in BSE behavior between academic majors. According to the IMB model (7), the adoption of health behavior is influenced by the interaction among three components: information, motivation, and behavioral skills. Our findings demonstrate the contribution of each construct to BSE behavior. Information (knowledge) was not directly associated with BSE behavior in the adjusted model, which is consistent with the theoretical assumption that knowledge may influence behavior primarily through motivation and behavioral skills. However, the lack of a significant direct association does not establish the presence of an indirect pathway because formal mediation analysis was not conducted in this cross-sectional study. Therefore, these findings are consistent with, but do not confirm, the IMB model's assumptions regarding the potential mediating roles of motivation and behavioral skills. Future longitudinal studies using formal mediation approaches, such as bootstrapped indirect effects or structural equation modeling, are needed to rigorously evaluate these pathways. Motivation (perceived barriers) emerged as the strongest predictor (Wald = 135.249). The finding that “not knowing the correct method” was the most frequently reported barrier (51.9%) highlights the importance of incorporating practical training into breast health education. Behavioral skills (self-efficacy) were significantly associated with BSE behavior (Wald = 31.977), suggesting that greater confidence in performing BSE may indicate behavioral initiation. Collectively, these findings support the IMB premise that effective behavior change requires addressing all three components simultaneously. The results of this study suggest that the higher BSE performance among nursing students may be associated with differences in information, motivation, and behavioral skills. Regarding the information component, nursing students achieved significantly higher BC prevention and control knowledge scores than pharmacy students (7.21 ± 2.51 vs. 6.05 ± 2.89, *p* < 0.001), although the effect size was small to medium (Cohen's d = 0.43), indicating a modest practical difference. For the motivation component, nursing students reported significantly lower perceived barrier scores than pharmacy students (2.65 ± 0.78 vs. 2.95 ± 0.85, *p* < 0.001, Cohen's d = −0.37). Regarding behavioral skills, nursing students exhibited significantly higher self-efficacy than pharmacy students (3.35 ± 0.72 vs. 3.18 ± 0.79, *p* < 0.001); however, the effect size was small (Cohen's d = 0.22), indicating limited practical significance. Among the three IMB constructs, perceived barriers showed the strongest independent association with BSE behavior in the multivariate model (Wald = 135.249), suggesting that it may represent an important target for future intervention research. However, this finding reflects a cross-sectional association and does not provide evidence that reducing perceived barriers directly improves BSE practice. Experimental or longitudinal intervention studies are needed to determine whether interventions targeting perceived barriers can lead to sustained improvements in BSE behavior. These findings are consistent with the core principles of the IMB model, which propose that information, motivation, and behavioral skills interact to influence health behavior. Compared with previous research, a study conducted at a university in Luzhou reported that female students in medical programs had a significantly higher BSE rate than their nonmedical counterparts, with this difference primarily attributed to their stronger foundation in medical knowledge (19). When interpreting cross-study comparisons, variations in participant characteristics (e.g., age and academic background), measurement instruments (e.g., self-reported vs. validated questionnaires), and cultural contexts (e.g., differences in health awareness and access to screening education) should be considered, as these factors may contribute to differences or similarities in reported BSE rates across studies. Consistent with these findings, a survey conducted in Tianjin showed that medical students had significantly higher levels of breast health knowledge than nonmedical students, accompanied by significant differences in BSE practice between the two groups (20). The multivariate binary logistic regression analysis further demonstrated that previous exposure to breast-related knowledge (OR = 4.642, 95% CI: 3.231–6.670) and higher self-efficacy (OR = 1.817, 95% CI: 1.453–2.272) were independently associated with higher odds of BSE performance, whereas higher perceived barriers (OR = 0.286, 95% CI: 0.226–0.360) were associated with lower odds of BSE behavior. After incorporating perceived barriers and self-efficacy into the regression model, the association between academic major and BSE behavior was attenuated, with the OR decreasing from 1.912 to 1.648. This finding suggests that the motivation and behavioral skills components of the IMB may partially explain the observed differences in BSE behavior between academic majors (21). However, because this study employed a cross-sectional design and did not conduct formal mediation analysis, these findings should be interpreted as observational associations rather than evidence of causal mediation. In the fully adjusted regression model controlling for knowledge, grade level, place of residence, and family history, each one-point increase in perceived barriers was associated with an approximately 71% reduction in the likelihood of performing BSE (adjusted OR = 0.286, 95% CI: 0.226–0.360). In contrast, each one-point increase in self-efficacy was associated with an approximately 1.82-fold increase in the likelihood of engaging in BSE (adjusted OR = 1.817, 95% CI: 1.453–2.272) after adjustment for the same covariates. These findings are consistent with the core principles of the IMB, which propose that information, motivation, and behavioral skills interact to influence health behavior. Nevertheless, given the cross-sectional design of this study, these associations cannot establish causal relationships pr confirm underlying mechanisms. Although individuals may possess adequate knowledge and behavioral skills, the adoption of health behaviors can still be impeded by perceived barriers, including embarrassment, forgetfulness, or unfamiliarity with the examination procedure. Therefore, medical colleges may consider implementing diverse approaches to breast health education, such as women's health lectures, peer-led workshops, and optional elective courses, rather than relying exclusively on compulsory coursework. This flexible approach can address differences in curricular arrangements across disciplines while providing students with accessible opportunities to acquire breast health knowledge and practical BSE skills. Numerous studies based on the IMB and other related theoretical frameworks have demonstrated significant associations between health beliefs and BSE behavior (15, 21). Overall, these findings indicate that health education interventions aimed at strengthening self-efficacy (behavioral skills) and reducing perceived barriers (motivation) may effectively promote BSE practice among students while improving BC-related knowledge and health beliefs (22, 23). In this cross-sectional study, previous exposure to breast-related knowledge was associated with higher odds of BSE performance (OR = 4.642, 95% CI: 3.231–6.670), and a higher total knowledge score was also associated with increased odds of BSE behavior (OR = 1.200, 95% CI: 1.127–1.279). However, because knowledge and behavior were assessed simultaneously, the temporal direction of this association cannot be determined. Students with a pre-existing interest in breast health may have been more likely to acquire relevant knowledge, rather than knowledge directly leading to increased BSE practice. Longitudinal studies are needed to clarify this temporal relationship. Self-efficacy (OR = 1.817) and perceived barriers (OR = 0.286) were identified as important factors that may contribute to the pathway linking knowledge with BSE behavior. Despite relatively high levels of knowledge, a substantial knowledge–behavior gap was observed. Among students who correctly identified that BSE should be performed monthly (K7, 83.8%), only 11.4% reported regular BSE, whereas 45.5% had never performed BSE. This gap between knowledge and practice was statistically significant (χ^2^ = 61.472, *p* < 0.001, Cramér's V = 0.246), indicating a moderate association between knowledge of BSE frequency and actual BSE behavior. Even among students with comprehensive knowledge of both the recommended frequency and optimal timing of BSE (K7 and K8 both correct, 69.7%), only 13.2% reported regular BSE practice, while 39.2% had never performed BSE. Similarly, among students with comprehensive knowledge of both the recommended frequency and optimal timing of BSE (K7 and K8 both correct, 69.7%), the regular BSE rate was only 13.2%, and 39.2% had never performed BSE. This gap, in which nearly nine out of ten knowledgeable students did not regularly practice BSE, highlights that knowledge alone is insufficient to maintain preventive health behaviors. Therefore, effective interventions should focus on strengthening health beliefs and improving behavioral skills. Previous studies have shown that virtual education delivered solely through social networking platforms may not be sufficient to improve all dimensions of BSE competencies. In particular, technical accuracy, including appropriate palpation patterns, pressure application, and examination of all breast quadrants, remains insufficiently addressed in virtual-only educational formats. In addition, these platforms may not adequately promote confidence in performing BSE or support sustained practice over time. For instance, Kucheki et al. (24) reported that a virtual educational intervention based on self-efficacy theory significantly improved BSE skills; however, the intervention primarily enhanced knowledge and self-reported behavior rather than directly ensuring long-term retention of technical proficiency. These findings suggest that although virtual education can effectively deliver information, standalone social media-based approaches may be insufficient for developing the psychomotor skills and sustained behavioral commitment required for competent BSE performance. Therefore, breast health education should extend beyond knowledge dissemination (information) and incorporate strategies targeting the other two IMB constructs. Hands-on training using breast models may enhance self-efficacy (behavioral skills), while peer education and experience-sharing activities may help reduce perceived barriers, such as embarrassment (motivation). This integrated approach addresses all three components of the IMB model and is consistent with the finding that perceived barriers were the strongest correlate of BSE behavior. Health promotion programs in colleges and universities should consider implementing the following strategies. Based on these findings, health promotion programs in colleges and universities may consider implementing the following strategies to address the identified barriers. First, because “not knowing the correct method” was the most commonly reported barrier (51.9%), accessible educational resources, such as simplified BSE procedure illustrations and brief instructional videos, should be developed to facilitate convenient and cost-effective learning. Furthermore, hands-on training using breast models is recommended to enable students to practice proper examination techniques in simulated environments, as our findings demonstrated that higher self-efficacy was independently associated with increased BSE performance. Second, the association between lower perceived barriers and regular BSE suggests that peer-led education initiatives may be beneficial. Engaging students who regularly perform BSE to share their experiences could foster a supportive and stigma-free communication environment, thereby reducing embarrassment-related barriers and promoting greater acceptance of BSE. Beyond these evidence-based recommendations, several additional strategies require evaluation in future interventional studies. Digital health tools, such as menstrual cycle tracking applications with integrated BSE reminders, represent a potential intervention strategy that warrants further investigation, as 25.7% of students reported forgetfulness as a barrier to regular BSE. However, these tools were not evaluated in this study, and their effectiveness remains uncertain. Future research should assess whether digital reminder interventions can improve not only BSE frequency but also technique accuracy, self-efficacy, and long-term adherence. Furthermore, culturally tailored interventions that address traditional norms related to female body privacy, which may influence BSE frequency, should be developed and rigorously evaluated through longitudinal or quasi-experimental designs before broader implementation.

In this study, family history of BC was not significantly associated with BSE behavior in the fully adjusted model (OR = 0.755, 95% CI: 0.466–1.222, *p*= 0.252). The wide confidence interval, which included both protective and risk directions, indicates limited precision of this estimate. This may be attributable to the relatively small number of participants with a positive family history (*n* = 101, 10.0%) and the resulting limited statistical power to detect a true association. In addition, interaction analyses between family history and IMB constructs, such as knowledge, perceived barriers, and self-efficacy, were not conducted. Family history may function as an effect modifier rather than solely as an independent predictor; for instance, the association between perceived barriers and BSE behavior may differ between students with and without a family history of BC. Therefore, this null finding should not be interpreted as definitive evidence of the absence of an association. Future studies with larger samples and greater representation of individuals with a family history of BC should evaluate both main effects and potential interactions to further clarify the role of family history in BSE behavior. The wide confidence interval, which included values suggesting both a potentially protective and a possible risk effect, indicates limited precision of the estimate, likely due to the low prevalence of a positive family history in our sample (10.0%, *n* = 101). Previous studies have reported inconsistent findings regarding this association. Some studies have identified a positive relationship, including research among Chinese female non-medical college students (25) and a 2026 umbrella review involving multiple low- and middle-income countries (OR = 1.81) (26). In contrast, a study among adolescents in the United States reported no significant association (27). These differences across populations suggest that the relationship between family history and BSE behavior may be context-dependent. Future studies with larger samples and greater representation of individuals with a family history of BC are needed to obtain more precise estimates and identify potential moderating factors.

Several limitations should be considered when interpreting the findings of this study. First, the cross-sectional design limits the ability to establish temporal relationships between exposure and outcome variables. Since knowledge, health beliefs, and BSE behavior were assessed simultaneously, it remains unclear whether knowledge and health beliefs influence subsequent BSE behavior or whether individuals who practice BSE are more likely to acquire relevant knowledge and develop strong health beliefs. Therefore, the findings should be interpreted as associations rather than causal relationships. Second, BSE behavior was assessed using self-reported data, which may be subject to recall bias and primarily reflects only whether students initiated BSE rather than the frequency, consistency, or technical competence of the practice. Because regular BSE practice was a key outcome of this study, this measurement limitation is particularly important. Students who reported having performed BSE may not have engaged in regular or technically correct practice. In related work from our group using a frequency-based classification, only 9.96% of students reported performing BSE at least once per month, suggesting that a considerable proportion of self-reported BSE may represent occasional rather than guideline-adherent practice. Future studies should incorporate validated behavioral measures that assess BSE frequency, adherence to recommended timing, and procedural accuracy to provide a more comprehensive evaluation of this preventive behavior. Social desirability bias may also have contributed to overreporting of this socially desirable health behavior. Third, participants were recruited from nursing and pharmacy programs at a single college, which may limit the generalizability of the findings to students from other academic disciplines or regions. Moreover, the inclusion of only healthcare-related majors may introduce selection bias because these students may have greater health awareness than students from nonmedical disciplines. Further studies involving broader and more diverse populations are required to confirm the results. Fourth, this study focused on self-reported BSE behavior rather than clinical screening outcomes, such as clinical breast examination (CBE) or mammography. Although self-reported BSE practice provides valuable information regarding individual preventive behaviors, it does not assess the quality or accuracy of the examination technique. In addition, objective measures, including clinical breast examinations and imaging-based screening, were not included in the assessment. Future studies could incorporate objective evaluations of BSE technique, such as standardized checklists or assessments by trained observers, as well as longitudinal follow-up of preventive behaviors to monitor changes over time and evaluate the effectiveness of educational interventions. Fifth, this study did not assess several potentially relevant factors, including clinical exposure, personal health awareness, family communication regarding breast health, and informal health learning through peers or social media. These unmeasured factors may differ systematically between nursing and pharmacy students. For instance, nursing students typically receive more clinical training and have greater direct patient contact, which may enhance their health awareness and reinforce preventive health behaviors through professional socialization. Such differences may confound the observed associations between academic major, IMB constructs, and BSE behavior, potentially resulting in an overestimation of the independent contribution of the measured IMB variables. Future studies should incorporate these factors to more comprehensively evaluate inter-major differences and minimize residual confounding. The absence of these variables may have resulted in residual confounding and influenced the interpretation of differences in BSE behavior between academic majors.

## 5 Conclusions

This cross-sectional study involving 997 female nursing and pharmacy students found that nursing students demonstrated higher BC prevention knowledge, lower perceived barriers, and higher self-efficacy than pharmacy students. Perceived barriers were the strongest correlate of BSE behavior (adjusted OR = 0.286), whereas self-efficacy (adjusted OR = 1.817) and prior breast health education (adjusted OR = 4.642) were independently associated with higher odds of BSE practice. Given the cross-sectional design, these associations should not be interpreted as causal relationships, and whether modifying these factors directly improves BSE behavior remains to be determined. These findings identify perceived barriers and self-efficacy as potentially modifiable correlates that can inform the development of future interventions. However, their causal effects on BSE behavior require confirmation through longitudinal or experimental studies. Notably, 51.9% of students identified “not knowing the correct method” as the primary barrier, and knowledge was not independently associated with BSE behavior after adjustment for other IMB constructs. Based on these findings, breast health education programs in medical colleges may benefit from incorporating two complementary components, although these strategies represent potential intervention approaches that require further validation. First, the observed associations suggest that information and motivation should be addressed concurrently by providing breast health knowledge while reducing perceived barriers, such as embarrassment, through peer education and experience-sharing activities. However, these strategies were not directly evaluated in this cross-sectional study, and their effectiveness remains to be established. Future research should use longitudinal or experimental designs to determine whether IMB-based educational programs incorporating knowledge dissemination, barrier reduction, and skills training can achieve sustained improvements in BSE practice. Future interventional studies are required to assess the effectiveness of this integrated approach. Second, the association between higher self-efficacy and BSE suggests that motivation and behavioral skills should be strengthened together. Previous intervention studies have shown that practical training using breast models can improve BSE technique accuracy and self-efficacy by providing hands-on practice with immediate feedback. Similarly, BSE diaries and self-monitoring tools have been associated with improved adherence to regular BSE practice, potentially by promoting habit formation and self-regulation. These approaches directly address the two most commonly reported barriers identified in our sample: not knowing the correct method (51.9%) and forgetfulness (25.7%). However, because these specific strategies were not directly evaluated in this study, their effectiveness among this population requires confirmation through future interventional research.

## Data Availability

The raw data supporting the conclusions of this article will be made available by the authors, without undue reservation.
